# Fall Direction Detection in Motion State Based on the FMCW Radar

**DOI:** 10.3390/s23115031

**Published:** 2023-05-24

**Authors:** Lei Ma, Xingguang Li, Guoxiang Liu, Yujian Cai

**Affiliations:** School of Electronic Information Engineering, Changchun University of Science and Technology, Changchun 130022, Chinayujiantsai@mails.cust.edu.cn (Y.C.)

**Keywords:** FMCW radar, fall direction detection, pattern feature extraction, dual-branch convolutional neural network

## Abstract

Accurately detecting falls and providing clear directions for the fall can greatly assist medical staff in promptly developing rescue plans and reducing secondary injuries during transportation to the hospital. In order to facilitate portability and protect people’s privacy, this paper presents a novel method for detecting fall direction during motion using the FMCW radar. We analyze the fall direction in motion based on the correlation between different motion states. The range–time (RT) features and Doppler–time (DT) features of the person from the motion state to the fallen state were obtained by using the FMCW radar. We analyzed the different features of the two states and used a two-branch convolutional neural network (CNN) to detect the falling direction of the person. In order to improve the reliability of the model, this paper presents a pattern feature extraction (PFE) algorithm that effectively eliminates noise and outliers in RT maps and DT maps. The experimental results show that the method proposed in this paper has an identification accuracy of 96.27% for different falling directions, which can accurately identify the falling direction and improve the efficiency of rescue.

## 1. Introduction

Fall accidents pose a major threat to the health and life of the elderly. When a fall accident occurs, the elderly person may be seriously injured or lose consciousness. It is necessary to detect falls in time and provide detailed fall information to help medical staff quickly formulate a treatment plan [1,2,3].

The directions of falls in motion can be categorized into four types: forward, backward, left, and right. Different fall directions can result in injuries to different parts of the body [4,5]. In reality, elderly individuals often lose consciousness and roll unconsciously after a fall. Upon arriving at the scene, medical staff are often faced with the daunting task of identifying the precise location of the injury, which can prove to be a challenging and time-consuming process, ultimately delaying the administration of prompt and effective treatment. Therefore, it is essential to conduct a specialized study on this subject. By detecting the precise direction of the fall, paramedics can receive valuable information to promptly formulate an effective treatment plan [6,7,8].

Researchers have explored different sensors for the detection and warning of fall accidents, such as wearable sensors and vision sensors [9,10,11,12,13,14,15]. In [16,17], the researchers used wearable sensors to detect the fall direction and determine the location of the fracture. In [18], the researchers proposed a relationship between the direction and severity of a fall during fall detection. They classified falls by using an accelerometer-based multi-classifier and trained five different models. In [19], the researchers investigated the correlation between fall direction and injury severity. They used the SisFall dataset and a support vector machine classifier to determine the direction and impact of falls. In [20], a further study of fall and non-fall actions was conducted, and a classification method was developed for falls of different directions and severities in the paper, which also included four common non-fall actions.

In [21], the researchers used camera equipment to extract gait differences between different movements to identify falls. They designed a multimodal feature fusion model that addresses the dependencies between spatial and temporal features in fall detection. The detection accuracy of the model was 95.80%. In [22], the researchers used camera equipment to detect the fall event and the posture after the fall. They designed a multi-scale skip connection segmentation network to obtain the human body contour from the camera. The accuracy of the model was 97.68%. In [23], the researchers utilized camera equipment to identify various human activities and enhanced the conventional long short-term memory network by introducing a new deep convolutional long short-term memory network (ConvLSTM). They determined the position of human bones from the images and combined motion features to create new features. The model achieved a detection accuracy of 98.89%. In [24], the researchers utilized a camera device to detect fall events. They employed the pose estimation method to acquire human joint information and used SVM for classification. Notably, this was the first instance of a visual sensor being employed to detect the direction of falls.

However, wearable sensors have limitations such as a high false alarm rate and inconvenience in terms of portability. Vision sensors have high light requirements and privacy leakage problems, particularly in private areas such as bathrooms or bedrooms [25,26]. Therefore, we selected the FMCW radar sensor as the data acquisition device [27]. The FMCW radar has the feasibility of all-weather monitoring, robustness in light changes, security in privacy protection, and the ability to perceive changes in speed and range.

The researchers analyzed the influence of human motion on the echo signal of the FMCW radar from different angles, such as the range–time (RT) feature, the Doppler–time (DT) feature, and the range–Doppler (RD) feature [28,29,30]. In [31], the researchers extracted the phase information from the intermediate frequency signal and obtained the distance change between the human head and eyes through this information. This method has a good effect on detecting small movements of the human body, such as blinking and micro head movements. Although a single feature can effectively identify human motion in most scenes, there are still problems with similar or indistinct features.

To address the issue of inadequate information in a single feature, researchers used a multi-feature fusion method to detect human motion. In [32], Branka Jokanovic et al. combined the time–frequency, time–range, and range–Doppler features. They used a deep neural network for fall detection. The experimental results showed that certain features were more beneficial than others in identifying specific motions. This further strengthens the advantages of motion classification that utilizes multiple features. In [33], Yuh-Shyan Chen et al. proposed the continuous human motion recognition (CHMR) algorithm. To distinguish highly similar human actions, they combined 2D features and 3D features, such as range, Doppler, angle, range–Doppler–time, and range–angle–time. In [34], Baris Erol et al. proposed a multi-linear subspace fall detection method. They used the slow time, fast time, and Doppler frequency features to construct a data cube for fall detection.

Subsequently, in [35], Feng Jin and others collected the point cloud and centroid information of the tested person and used a Hybrid Variational RNN Autoencoder for fall detection. In [36], Xingshuai Qiao et al. proposed a radar point clouds model. They used range–time, Doppler–time, and Doppler–range to construct an RPC cube and used a two-layer CMPCANet to detect fall events. In [37], Ahmed Zoha et al. used the micro-Doppler features of the FMCW radar to detect falls. In addition, they used a variety of machine learning algorithms and transfer learning algorithms to classify human actions.

Although the above studies have shown that fall events can be effectively identified through the various features of the FMCW radar, there has been less work on detecting and identifying the direction of falls in motion. Based on the aforementioned analysis, this study utilizes the FMCW radar to detect the direction of falls in motion among elderly individuals. We combine range–time features and Doppler–time features and use a dual-branch CNN to achieve fall direction detection and recognition. To improve the model’s reliability and reduce the system’s computational cost, this paper proposes a pattern feature extraction (PEF) algorithm to eliminate noise and outliers in the environment.

The rest of this paper is structured as follows. Section 2 introduces the Materials and Methods. Section 3 introduces the Experimental Platform. Section 4 provides the Experimental Results. Section 5 provides the Conclusions, summarizing the research conducted and their implications for future research in the field.

## 2. Materials and Methods

### 2.1. Fall Direction Detection System

**Figure 1:**
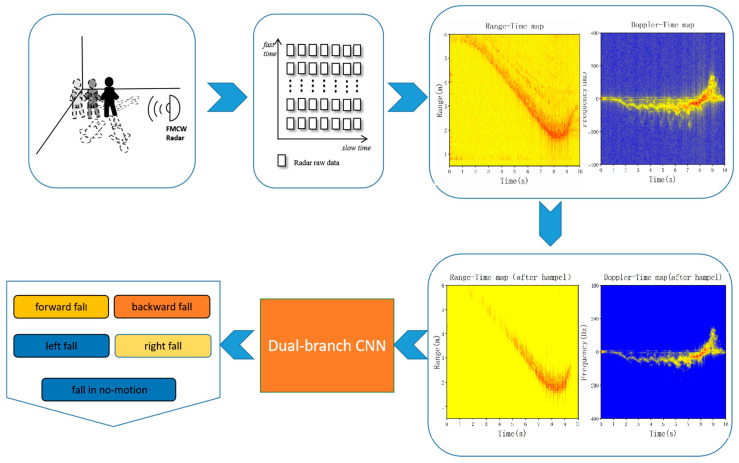
Fall direction detection system.

The fall direction detection system we designed is depicted in Figure 1. We collected human motion information using the FMCW radar and used a signal processing algorithm to process the original radar information to obtain the range change and Doppler change from the motion state to the falling state. We use the pattern feature extraction (PEF) algorithm to remove noise and outliers in the environment and to feed the processed data into the built dual-branch CNN to identify the direction of the fall.

### 2.2. Radar Raw Data Processing

**Figure 2:**
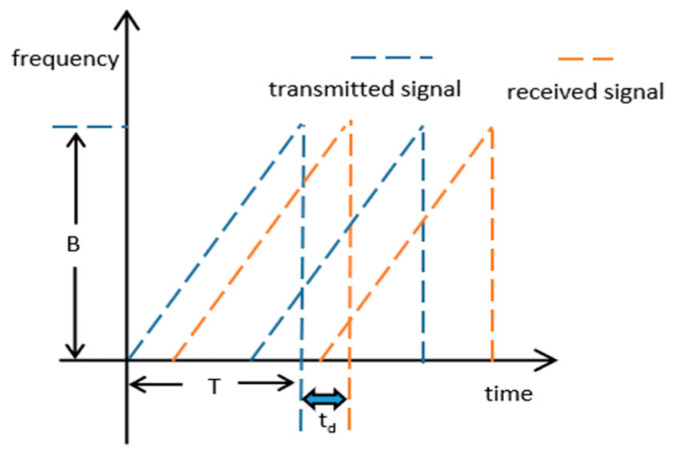
The frequencies of the signal waveform.

When the FMCW radar is working, the transmit and receive antennas are switched on synchronously. The frequencies of the transmitted and received signals are depicted in Figure 2. The frequencies of the signal waveform. The transmitted signal can be written as

(1) $$x_{T}\left(t\;\right)=A_{T}\cos\left(2\pi f_{c}+\pi \frac{B}{T_{c}}t^{2}+\varphi \left(t\right)\right),$$

where $A_{T}$ is the transmitting power, $\varphi \left(t\right)$ is the phase noise of the transmitter, $f_{c}$ is the initial frequency of the chirp; B is the bandwidth of the chirp and $T_{c}$ is the duration of the chirp.

The point target within the effective range of the radar will generate a reflected signal which the radar receives after a brief delay. The delay can be written as

(2) $$t_{d}=2d/c,$$

where d is the distance of the measured target. The signal that the radar receives can be written as:

(3) $$x_{R}\left(t\right)=\alpha A_{T}cos\left[2\pi f_{c}\left(t-t_{d}\right)+\pi \beta {\left(t-t_{d}\right)}^{2}\right],$$

where α is the factor of influence amplitude change caused by the attenuation of the environment and the scattering of the object. The specific formula for the beat frequency signal $f_{b}$ can be written as:

(4) $$f_{b}=\beta \cdot t_{d},$$

where β is the slope of the chirp signal. We can obtain baseband I/Q signals and then obtain complex baseband data $Z\left(n\right)$ through the A/D converter.

**Figure 3:**
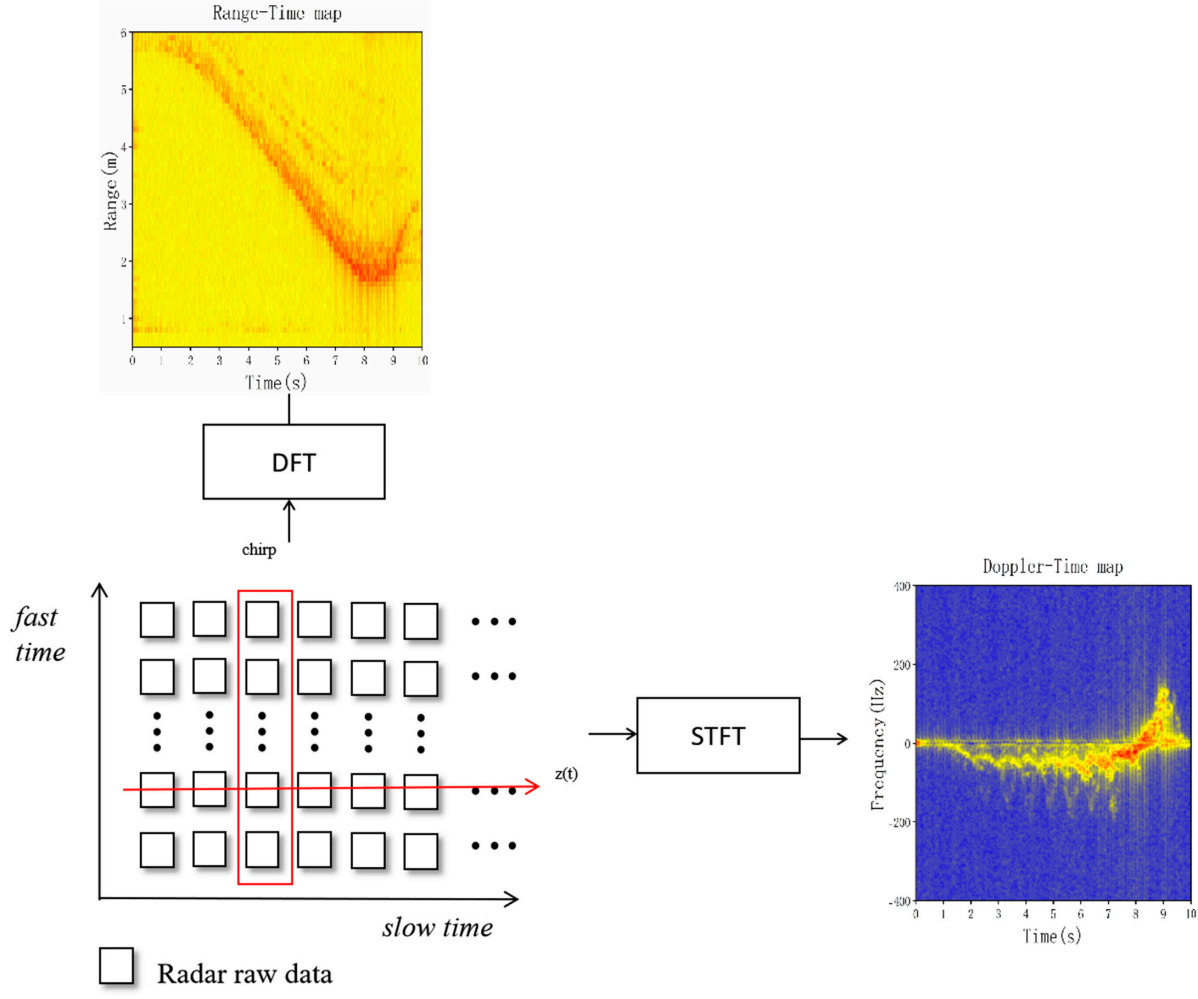
The radar baseband data matrix generates the RT map and the DT map.

When all chirps are ordered by time, a comprehensive matrix of radar raw data is generated, accurately capturing critical information. The matrix is crafted with horizontal and vertical axes, the former representing the slow time dimension and the latter representing the fast time dimension. As shown in Figure 3, we used the STFT algorithm to transform the slow time dimension of $Z\left(n\right)$ to obtain the DT characteristics. The specific formula for STFT can be expressed as

(5) $$STFT\left[z\left(t\right)\right]=s\left(\tau ,f_{d}\;\right)=\int_{-\infty }^{\infty }z\left(t\right)\omega \left(t-\tau \right)e^{-j2\pi f_{d}t}dt,$$

where $z\left(t\right)$ is the radar echo signal, $\omega \left(t\right)$ is the sliding window function, and $f_{d}$ represents the Doppler frequency. The distance equation of the measured object can be written as:

(6) $$d=\left(c\cdot f_{b}\right)/2\beta ,$$

We used the DFT algorithm to transform the fast time dimension of $Z\left(n\right)$ to obtain the RT characteristics. The specific formula for DFT can be written as:

(7) $$DFT_{N}\left(x\left[n\right]\right)=\sum_{k=0}^{N-1}x\left[n\right]e^{\frac{-j2\pi kn}{N}},$$

where $x\left[n\right]$ is the original signal.

**Figure 4:**
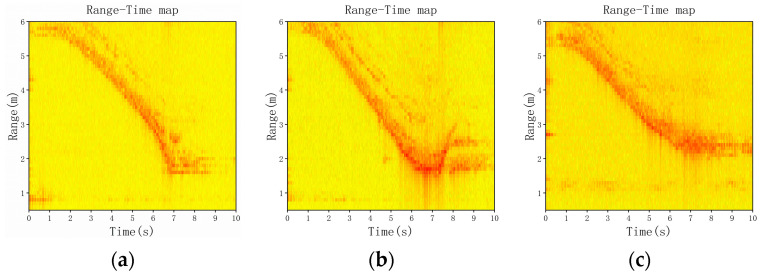

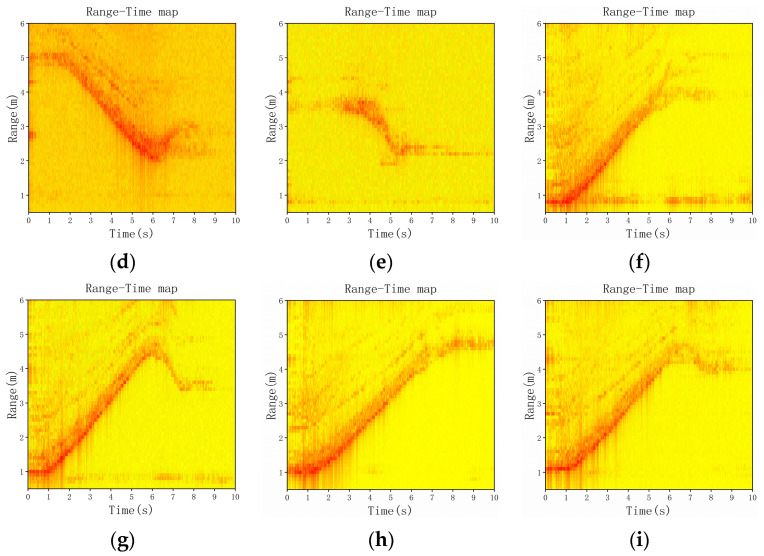
The RT maps of the different falling directions. (**a**) Forward fall in motion close to the radar, (**b**) backward fall in motion close to the radar, (**c**) left fall in motion close to the radar, (**d**) right fall in motion close to the radar, (**e**) fall in non-motion, (**f**) forward fall in motion away from the radar, (**g**) backward fall in motion away from the radar, (**h**) left fall in motion away from the radar, and (**i**) right fall in motion away from the radar.

**Figure 5:**
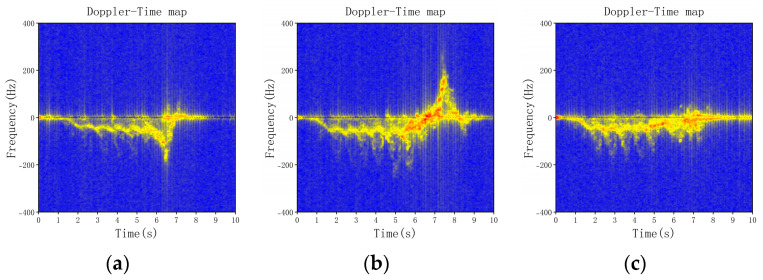

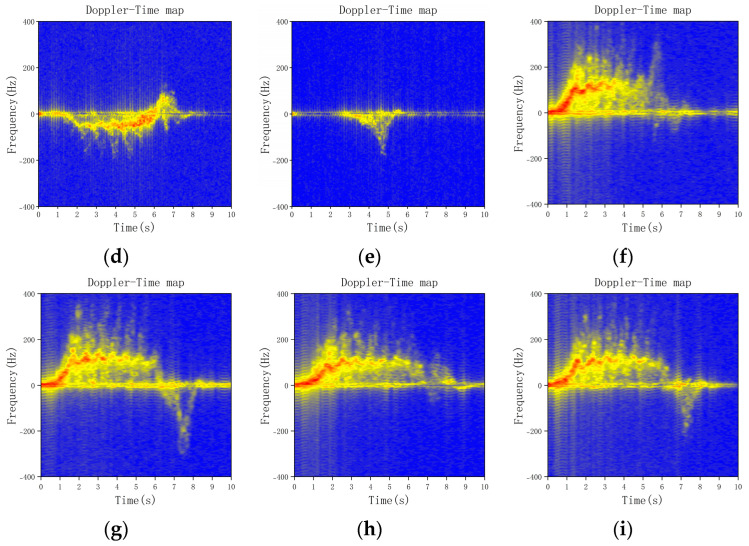
The DT maps of different falling directions. (**a**) Forward fall in motion close to the radar, (**b**) backward fall in motion close to the radar, (**c**) left fall in motion close to the radar, (**d**) right fall in motion close to the radar, (**e**) fall in non-motion, (**f**) forward fall in motion away from the radar, (**g**) backward fall in motion away from the radar, and (**h**) left fall in motion away from the radar, (**i**) right fall in motion away from the radar.

To improve the reliability of the experiment, we merged the four falling directions when close to the radar and the four falling directions when away from the radar into four falling directions in motion. Figure 4 shows the range–time map of different fall states. In addition, Figure 5 shows the Doppler–time map of different fall states.

From the range–time diagram in Figure 4, we can observe that when a person falls forward or to the left during motion, the distance from the motion state to the fallen state follows a similar trend. When approaching the radar, the distance between the person and the radar increases. In addition, when moving away from the radar, the distance between the person and the radar decreases. However, the change in distance for a forward fall is greater than that for a left fall. On the other hand, when a person falls backwards or to the right during motion, the distance from the motion state to the fall state changes in the opposite direction. When approaching the radar, the distance between the person and the radar first decreases and then increases. In addition, when moving away from the radar, the distance between the person and the radar first increases and then decreases. However, the change in distance for falling backwards is greater than for falling to the right. Therefore, we can identify different fall directions based on the range–time features.

From the Doppler–time graph in Figure 5, we can observe that when a person falls forward or to the left during motion, the Doppler change trend from the moving state to the falling state is the same. When approaching the radar, the Doppler information is negative. In addition, when far away from the radar, the Doppler information is positive. However, the Doppler change for a forward fall is greater than that for a left fall. On the other hand, when a person falls backward or to the right during motion, the Doppler change from the motion state to the fallen state is the opposite. When approaching the radar, the Doppler information is first negative and then positive. In addition, when far away from the radar, the Doppler information is first positive and then negative. However, the Doppler change for a backward fall is greater than that for a rightward fall. Therefore, we can identify different falling directions based on the Doppler-time features.

In summary, both range–time features and distance–time features can differentiate the direction of falls. However, to enhance the reliability of the model, we adopted a fusion of the two features for the recognition of fall direction.

### 2.3. Pattern Feature Extraction

**Figure 6:**
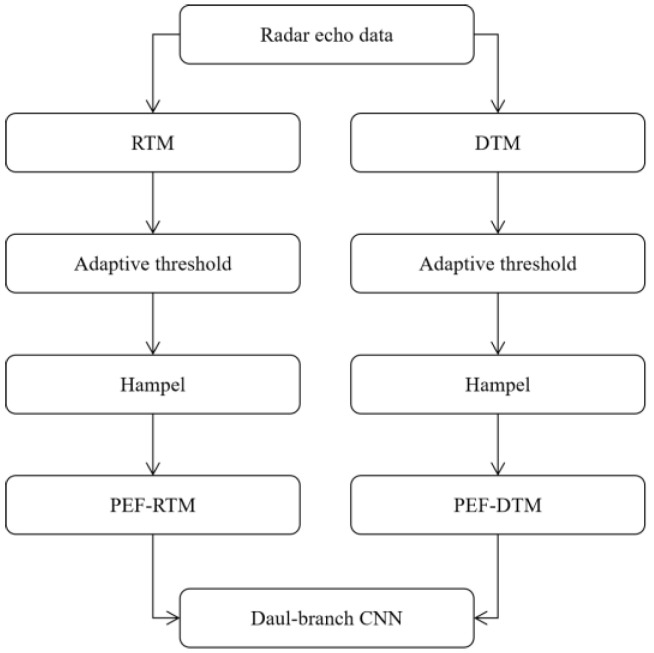
The proposed pattern feature extraction algorithm.

We can observe that there is a lot of noise and many outliers in the RT map and the DT map, which cause great interference in identifying the falling direction. Therefore, we propose a pattern feature extraction (PEF) algorithm to lessen computing costs and enhance the reliability of the model. Figure 6 shows the algorithm flowchart.

Different power values are chosen based on different feature maps. Regions in the feature matrix with high power values are selected. Points with power values higher than the threshold are considered valid feature regions, and the threshold formula can be written as:

(8) $$P_{th}=\left(1-a\right)\bar{P}+a\cdot \left\{\max\left\{P\right\}+min\left\{P\right\}\right\},$$

where $a\in \left(0,1\right)$ is an adaptive parameter. The highest power value, lowest power value, and average power value in the feature diagram are represented by $\max\left\{P\right\}$, $min\left\{P\right\}$, and $\bar{P}$, respectively. When creating a dataset, the power values in each feature map vary. Choosing the appropriate threshold for each feature image can reduce the ambient noise more effectively.

After choosing an appropriate threshold, there are still some discrete outliers in the feature map. Then, by removing the outliers by using the Hampel filter, the calculation formula of the Hampel filter can be written as:

(9) $$m_{k}=median\left\{x_{k-K},\ldots x_{k},\ldots x_{k+K}\right\}$$

(10) $$S_{k}=1.4826\times median\left\{\left|x_{k-j}-m_{k}\right||j\in \left[-K,K\right]\right\};$$

(11) $$\;\;\;\;\;\;\;\;\;\;\;\;y_{k}=\left\{\begin{array}{c} x_{k},\left|x_{k-j}-m_{k}\right|\leq n_{th}S_{k}\\ m_{k},\left|x_{k-j}-m_{k}\right|\leq n_{th}S_{k} \end{array}\right.$$

The median $\left\{\cdot \right\}$ is the median of the sought signal, and $n_{th}$ is the final threshold.

**Figure 7:**
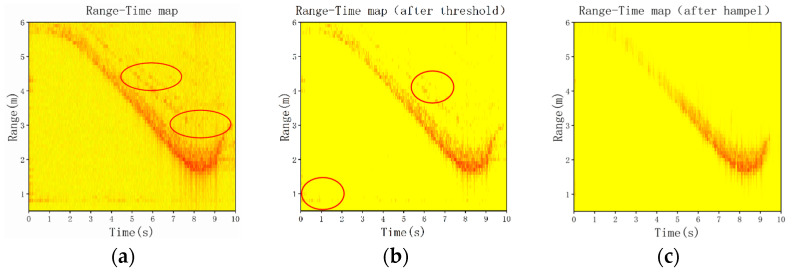

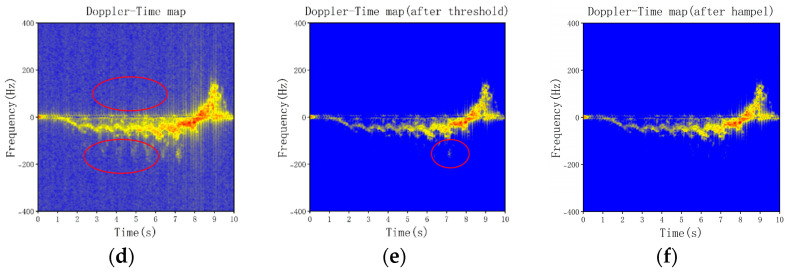
(**a**) RT map of backward fall in motion; (**b**) RT map after threshold of backward fall in motion; (**c**) RT map after Hampel of backward fall in motion; (**d**) DT map of backward fall in motion; (**e**) DT map after threshold of backward fall in motion; and (**f**) DT map after Hampel of backward fall in motion.

Figure 7a,d is the unprocessed RT map and the DT map. The area marked by the red circle is the noise from the surrounding environment. Figure 7b,e shows the RT map and the DT map with less noise and stronger features after processing with the power-threshold algorithm. The red circles in Figure 7b,e represent the remaining outliers. Figure 7c,f shows the results processed by the Hampel filter, which are more suitable as input for the CNN to improve the reliability of the model.

### 2.4. Dual-Branch CNN

The convolutional neural network (CNN) is a cutting-edge technology that utilizes internal convolution to extract a wide range of input features, including target color, edges, corners, and other critical information. These features serve as the foundation for CNN classification, allowing for unparalleled accuracy and precision in a variety of applications. By reducing the computational cost of preprocessing, CNN has become a go-to solution for classification tasks across a broad range of industries and disciplines. To identify different falling directions, it is necessary to detect changes in distance and Doppler before and after the person falls. Therefore, this experiment uses the RT map and the DT map as the input for the dual-branch CNN. The dual-branch CNN used in the experiments is traditional and basic. We are committed to using the simplest network structure to realize the recognition of the direction of the fall.

**Figure 8:**
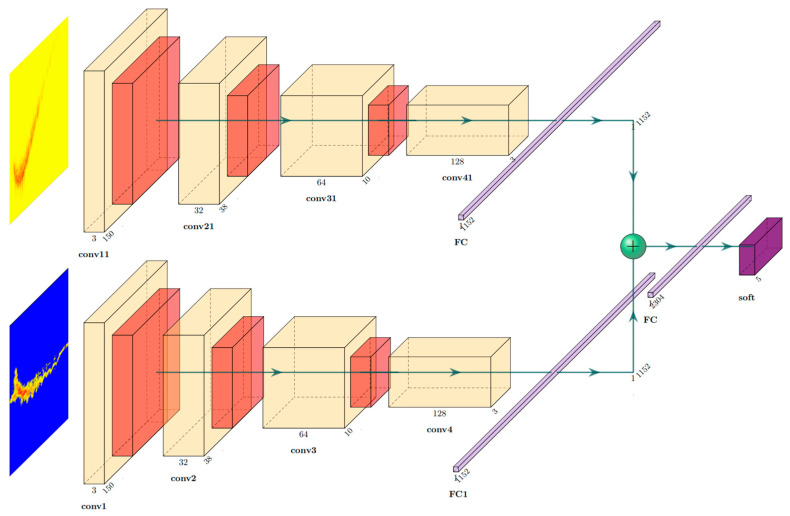
Dual-channel CNN (yellow is the convolutional layer, red is the max pooling layer, light purple is the fully connected layer, and purple is the soft classifier).

The network can fuse motion information from both the RT map and the DT map. In Figure 8, the network model of the dual-branch CNN is shown. Table 1 shows the parameter settings of each convolutional layer, pooling layer, and fully connected layer.

## 3. System Implementation Experiment Platform

### 3.1. Experiment Platform

The FMCW radar in the experiment is an IWR6843 produced by Texas Instruments (Dallas, TX, USA) [38]. The FMCW radar operates from 60 to 64 GHz. In our experiments, the radar is started at 60 GHz with a chirp slope of 33 MHz/μs. The number of sampling points selected on each chirp is 256. The other radar parameters are shown in Table 2.

### 3.2. Experimental Environment

**Figure 9:**
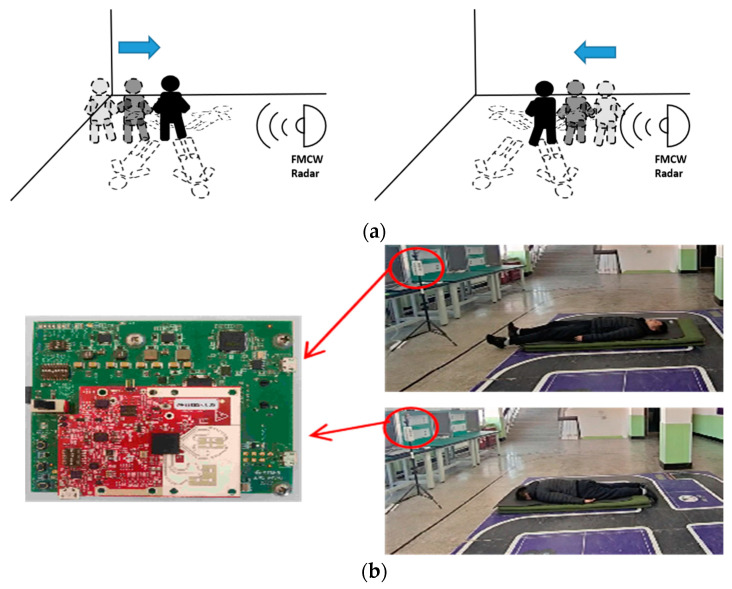
(**a**) Schematic diagram of different falling directions and (**b**) the experimental scene setup (**right**) and the FMCW radar (**left**).

The experimental environment setup is shown in Figure 9. The radar sensor is installed approximately 6 m in front left of the volunteer, with a height of about 1.5 m above the ground and inclined downward at 15° to ensure that the radar can collect the overall information. The experiment considers five fall states, including forward fall in motion, backward fall in motion, left fall in motion, right fall in motion, and fall in non-motion.

**Figure 10:**
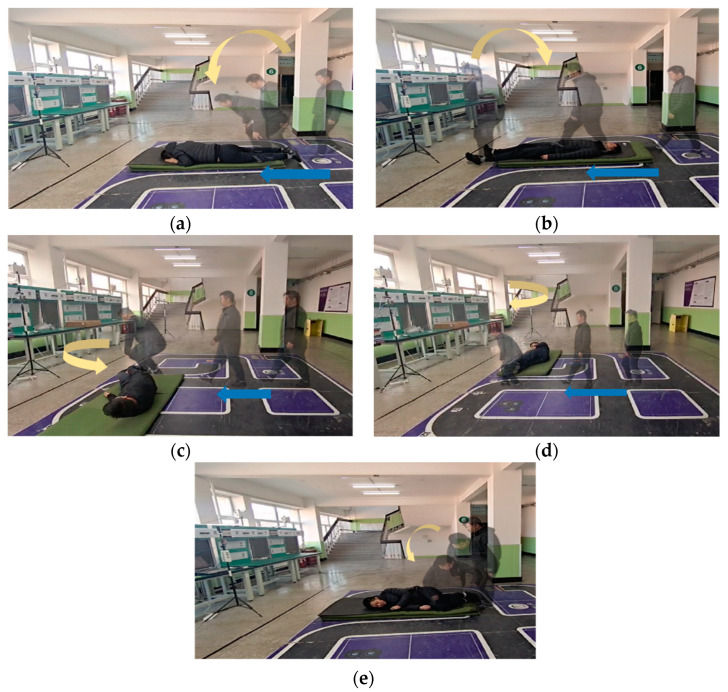
(**a**) Forward fall in motion (**b**) backward fall in motion, (**c**) left fall in motion, (**d**) right fall in motion, and (**e**) fall in non-motion.

Figure 10 shows the data collection scene for some actions in the experiment. The blue arrow in the figure represents the motion direction of the volunteers, while the yellow arrow represents the fall direction of the volunteers.

### 3.3. Data Collection

**Figure 11:**
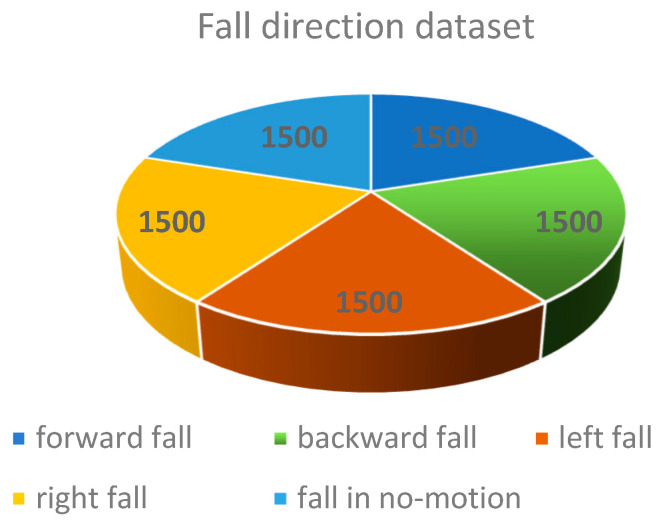
Data samples corresponding to each action.

Six volunteers participated in the experiment. The FMCW radar collected each action for 10 s. Each volunteer performed five actions. In addition, each movement was performed 80–100 times. A total of 573 pieces of data were collected. The experiment used the data sample generation method [39], and the radar mode was set to one transmission and four receptions. Through this method, we expanded the dataset to 7500 samples. Figure 11 is the proportion of data collected. Each color in the figure corresponds to a fall direction category. Each share is 1500 datasets.

## 4. Results

**Figure 12:**
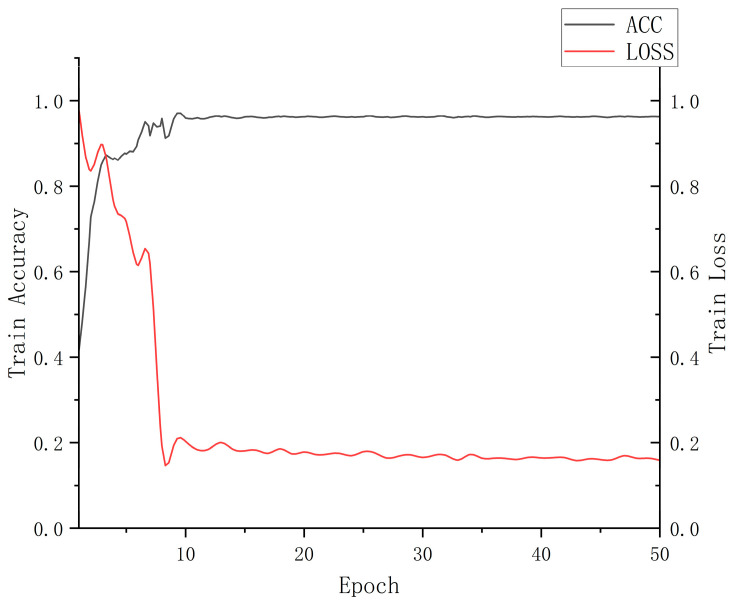
Accuracy and loss curves.

This experiment uses the Pytorch framework. The learning rate of the CNN is 0.0005, and the Step LR learning rate update strategy was used. The number of iterations is 50. Figure 12 shows the changes in accuracy and the loss of the dual-branch CNN during the training process. It can be observed that after 10 rounds of training, the network model tended towards a stable state. In addition, after 20 rounds, the model’s accuracy reaches its peak.

**Figure 13:**
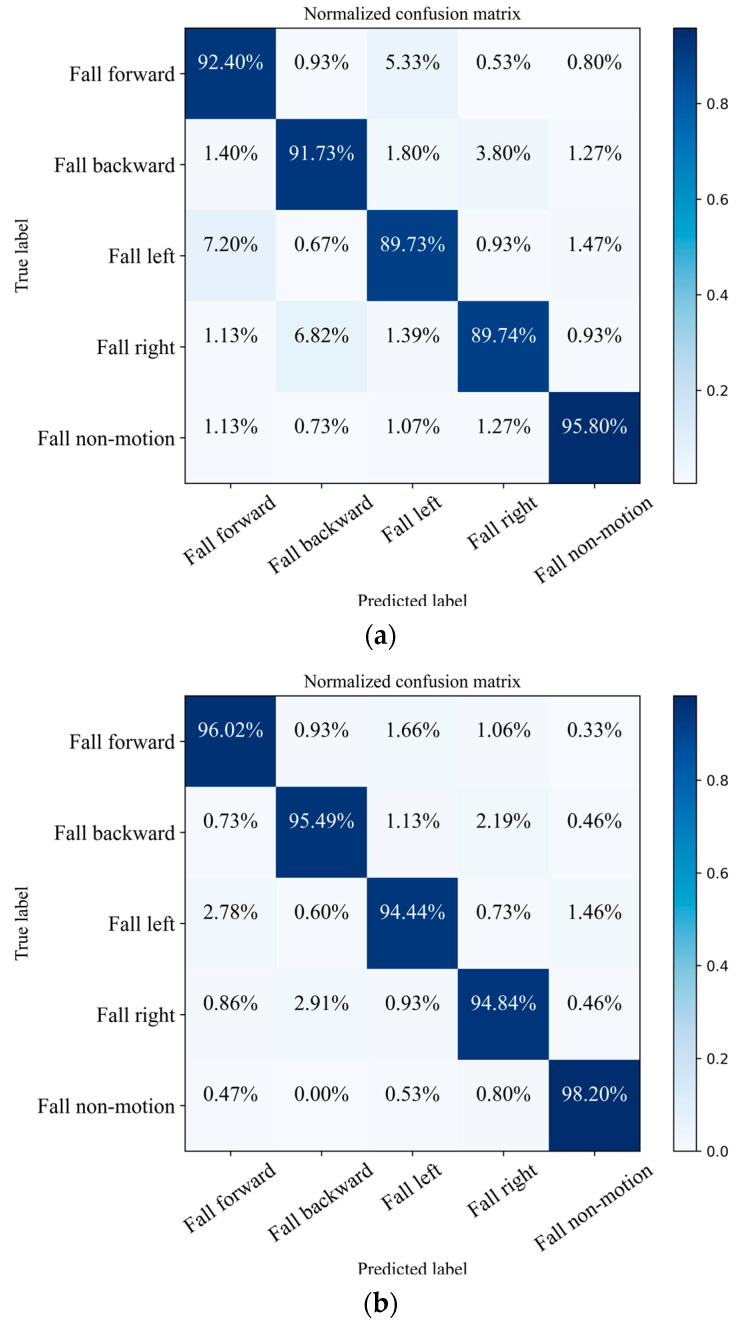
(**a**) Confusion matrix using the original dataset; (**b**) confusion matrix using the PEF dataset.

In order to comprehensively evaluate the efficacy and accuracy of our proposed PEF algorithm, we utilized both the unaltered original dataset and the refined PEF dataset to detect the fall direction. Figure 13a shows the confusion matrix using the original dataset as input in the dual-branch CNN. In addition, Figure 13b shows the confusion matrix using the PEF dataset as input in the dual-branch CNN.

The results of our experimentation reveal that when using the original dataset, the network’s recognition accuracy rates for forward fall in motion, backward fall in motion, left fall in motion, right fall in motion, and fall in non-motion were 92.40%, 91.73%, 89.73%, 89.74%, and 95.80%, respectively. When using the PEF dataset, the network’s recognition accuracy rates for forward fall in motion, backward fall in motion, left fall in motion, right fall in motion, and fall in non-motion were 96.02%, 95.49%, 94.44%, 94.84%, and 98.20%, respectively. The dual-channel CNN built in this experiment has a recognition rate of 96.27% for the direction of the fall. Our proposed PEF algorithm improves the accuracy of the model by 3–4%.

Forward fall and left fall have the same change trend in the distance, but the change in the distance is different. In order to analyze the influence of the height of the tested person on the experimental results, we conducted experiments using the respective datasets of six volunteers. Table 3 is the height of the tested person and the accuracy of the corresponding model.

The experimental results show that the taller the subject is, the easier it is to recognize the direction of the fall. This may be because the height of the person affects the distance change during the fall. The larger the distance variation, the more distinct features we acquire.

In order to further evaluate the superiority of the PEF algorithm, we chose CNNs with different structures for fall direction detection on the PEF dataset and the original dataset, including the two-branch CNN, LeNet [40], AlexNet [41], and VGG [42], and the DT map or the PEF-DT map as the input to the single-branch network. Table 4 and Table 5 are the falling direction detection results of different CNNs.

The experimental results show that the recognition of the fall direction by dual-branch CNN detection is higher than that of the other three single-branch networks. This is because its input is composed of distance characteristics and Doppler characteristics, which contain more human motion information. When using the PEF dataset, LeNet, AlexNet, and VGG show better results in terms of precision and recall. The results show that the PEF algorithm also performs excellently in CNNs with different structures.

## 5. Discussion

In this section, we review related works on fall orientation detection and discuss their methods and results. Table 6 shows the results of using different sensors and models in the field of identifying different fall directions.

In [16], Farhad Hossain et al. utilized a single 3D accelerometer to detect forward fall, backward fall, left fall, right fall, and ADL. They employed several machine learning algorithms for analysis and demonstration. The experimental results showed that the classification results using the SVM algorithm were the most accurate. The accuracy of the model was 94.54%. In [17], Abbas Shah Syed et al. used 3D accelerometers and gyroscopes to detect forward fall, backward fall, and lateral fall. In addition, they divided the fall action in each direction into two categories: hard fall and soft fall. They proposed the XGB-CNN network. The accuracy of the model was 90.02%.

In [18], Ryan M. Gibson et al. employed accelerometers to identify seven types of fall events, including forward fall, backward fall, left fall, right fall, hard fall, soft fall, and fall in any situation. They used the CVM algorithm to conduct experiments, and the experimental results showed that the accuracy of the model was 98.3%. In [19], Abbas Shah Syed et al. utilized the IMU-sensor-collected SisFall dataset to determine the direction of falls. They used the inertial measurement sensor and SVM algorithm to identify falls in three directions: forward, backward, and lateral. The model accuracy for fall detection was 90.4%.

In [24], Chunmiao Yuan et al. introduced a novel video-based method for detecting the direction of falls. They calculated human body joint points using body posture and identified four types of falls (forward, backward, left, and right) using the SVM algorithm. The model achieved an accuracy of 97.52% on the Le2i dataset.

However, there are some limitations to this paper. Specifically, we only employed a basic two-branch convolutional neural network to detect the direction of falls. In future research, we aim to develop a better performing network architecture that improves the overall performance of the system while improving accuracy.

## 6. Conclusions

This paper presents a fall direction detection method in motion based on the FMCW radar. In the study, we extract the range–time features and Doppler–time features of people in motion and fall states through the FMCW radar to distinguish different fall directions. We propose a PEF algorithm to reduce noise and outliers in feature maps. We built a dual-branch CNN to detect different fall directions and analyzed the performance of the PEF algorithm in different network structures. This method achieves high accuracy in identifying different fall directions.

In future work, we will collect fall direction data from volunteers of different ages to make our dataset more diverse. Additionally, we plan to prompt the injured body part and the corresponding treatment method after detecting the direction of the fall. This will enable elderly individuals to receive quick and effective treatment, reducing the risk of secondary injuries.

## Figures and Tables

**Table 1:** Dual-channel CNN structure.

Layer	Channel 1	Channel 2
Input	PEF-RT map	PEF-DT map
Convolution	(3, 16, 3)	(3, 16, 3)
Max Pooling	(2, 2)	(2, 2)
Convolution	(16, 32, 3)	(16, 32, 3)
Max Pooling	(2, 2)	(2, 2)
Convolution	(32, 64, 3)	(32, 64, 3)
Max Pooling	(2, 2)	(2, 2)
Convolution	(64, 128, 3)	(64, 128, 3)
Flatten	1×1152	1×1152
Convolution	1×2304	
Sigmoid	-	

**Table 2:** Rader parameters.

Parameters	Quantity
Starting frequency	60 GHz
Stop frequency	63.015 GHz
Bandwidth	3.015 GHz
Frequency slope	50.259 MHz/μs
Sample rate	5 MHz
Idle time between chirps	100 s
Ramp end time	60 s
Number of receiving antennas	4
Number of transmitting antennas	1
Frame time	10 s
Number of samples per chirp	256
No. of chirp loops	128

**Table 3:** The recognition accuracy of different heights.

Volunteer ID	Height (cm)	Accuracy
1	185	96.82%
2	181	96.55%
3	177	96.02%
4	175	95.86%
5	160	95.75%
6	158	95.75%

**Table 4:** Classification matrix using the original dataset.

Net	ACC	TPR- Forward Fall	TPR- Backward Fall	TPR- Left Fall	TPR- Right Fall	TPR-Fall Non-Motion
Two-Branch CNN	92.00%	92.40%	91.73%	89.73%	89.74%	95.80%
LeNet	87.72%	88.53%	88.33%	87.73%	87.26%	86.73%
AlexNet	88.96%	90.50%	89.10%	88.00%	89.26%	87.86%
VGG	89.89%	91.60%	90.53%	88.87%	89.87%	88.60%

**Table 5:** Classification matrix using the PEF dataset.

Net	ACC	TPR- Forward Fall	TPR- Backward Fall	TPR- Left Fall	TPR- Right Fall	TPR-Fall Non-Motion
Two-Branch CNN	96.27%	96.02%	95.49%	94.44%	94.84%	98.20%
LeNet	93.52%	93.86%	93.46%	91.73%	92.53%	96.00%
AlexNet	92.64%	92.13%	93.26%	91.73%	91.26%	94.80%
VGG	94.86%	93.46%	94.46%	94.53%	94.40%	97.46%

**Table 6:** Comparison of different fall direction detection methods.

References	Sensor Type	Model	Accuracy
[16]	Three-dimensional accelerometer	SVM	94.54%
[17]	Three-dimensional accelerometer and gyroscope	XGB-CNN	90.02%
[18]	Accelerometer	SVM	98.3%
[19]	IMU sensor	SVM	90.4%
[24]	Camera	SVM	97.52%

## Data Availability

Not applicable.
